# A recurrent homozygous *NHLRC1* variant in siblings with Lafora disease

**DOI:** 10.1038/s41439-018-0015-9

**Published:** 2018-07-12

**Authors:** Nami Araya, Yukitoshi Takahashi, Masayuki Shimono, Tomofumi Fukuda, Mitsuhiro Kato, Mitsuko Nakashima, Naomichi Matsumoto, Hirotomo Saitsu

**Affiliations:** 1 National Epilepsy Center, Shizuoka Institute of Epilepsy and Neurological Disorders, NHO, Shizuoka, Japan; 2 Department of Pediatrics, School of Medicine, University of Occupational and Environmental Health, Fukuoka, Japan; 3 Department of Pediatrics, School of Medicine, Showa University, Tokyo, Japan; 4 Department of Human Genetics, Graduate School of Medicine, Yokohama City University, Yokohama, Japan; 5 Department of Biochemistry, Hamamatsu University School of Medicine, Hamamatsu, Japan

## Abstract

We report a case of two siblings with progressive myoclonus epilepsy whose parents were not consanguineous. Their clinical symptoms were typical of Lafora disease (LD), but skin biopsies revealed no Lafora bodies. Whole-exome sequencing identified a recurrent homozygous frameshift variant in the *NHLRC1* gene in both siblings. The genetic analysis was useful for the diagnosis of LD, as neither consanguinity nor Lafora bodies were found.

Lafora disease (LD, OMIM # 254780) is one of progressive myoclonus epilepsy (PME) with a mode of autosomal recessive inheritance. The symptoms usually start in the teenage years, and development is normal before the onset of seizures. The electroencephalogram (EEG) shows occipital spike-and-slow waves in the early stage of the disease. The occipital seizures with visual onset symptoms and myoclonus are easily induced by intermittent photic stimulation. Mental dysfunction and ataxic movement progress rapidly in a year^1^. *EPM2A* and *NHLRC1* (*EPM2B*, RefSeq accession number NM_198586.2) are well-known causative genes for LD. More than 80% of LD patients carry variants in either gene; however, there is a possibility that another candidate gene may be involved in LD pathogenesis because some LD patients lack variants in either of them^2,3^. In the Japanese population, variants in *NHLRC1* are the most frequent cause of LD^4^. Here, we report two Japanese siblings with LD carrying a homozygous variant in the *NHLRC1* gene.

The proband (Fig. 1a; III-4) was born as the second child of unrelated Japanese parents. Her father was born in a town of northern Kyusyu and was healthy. Her mother was born in another town of northern Kyusyu and had a history of thyroid cancer. The patient had been a smart student until she suffered a generalized tonic clonic convulsion (GTC) at the age of 12 years. The initial symptom preceding GTC was visual hallucination. EEG showed occipital sporadic spikes and continuous slow waves in the bilateral occipital regions (Fig. 2a). She sometimes suffered visual hallucinations as a seizure manifestation and developed eyelid myoclonus. Six months after her first seizure, she experienced a second GTC and was diagnosed as having idiopathic occipital epilepsy with secondary generalization. She was prescribed carbamazepine but began to experience weekly GTCs and eyelid and generalized myoclonic seizures. Her cognitive and motor functions declined rapidly.

**Fig. 1:**
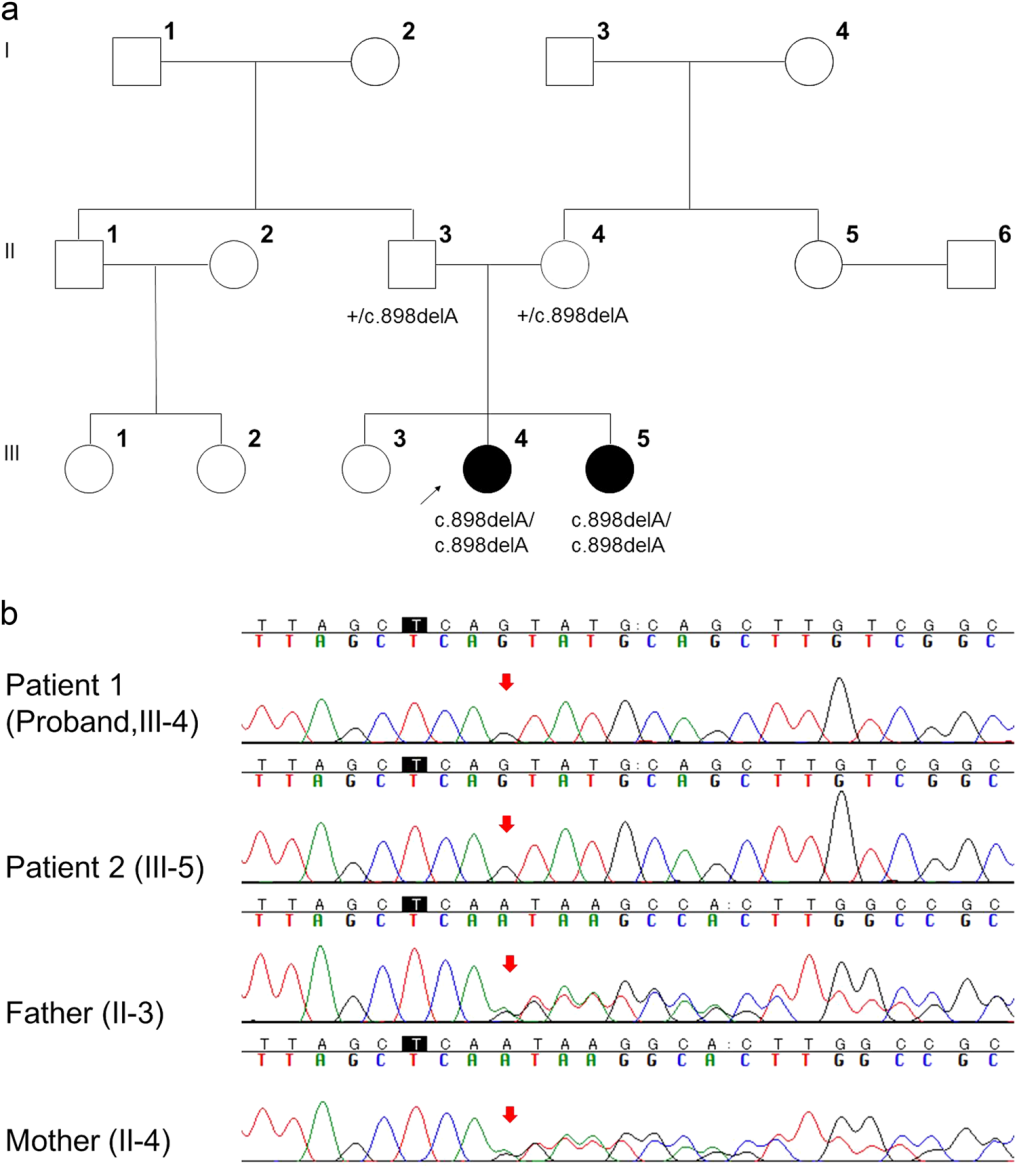
Pedigree tree and genetic analysis of Lafora disease family. **a** Pedigree tree of the LD family; III-4 (black arrow) is the proband. Her younger sister (III-5) also showed similar symptoms when she was 11 years old. Filled symbols represent individuals with LD. Genotypes of the *NHLRC1* variants are shown below each tested individual. Plus sign (+) indicates wild type. **b** Electropherograms of the proband (III-4), her younger sister (III-5), and their parents (II-3, 4). The proband and her younger sister had a homozygous NM_198586.2 (NHLRC1):c.898del: p. [Ser300Valfs*13] variant. Their parents had a heterozygous NM_198586.2 (NHLRC1):c.898del: p. [Ser300Valfs*13] variant

**Fig. 2:**
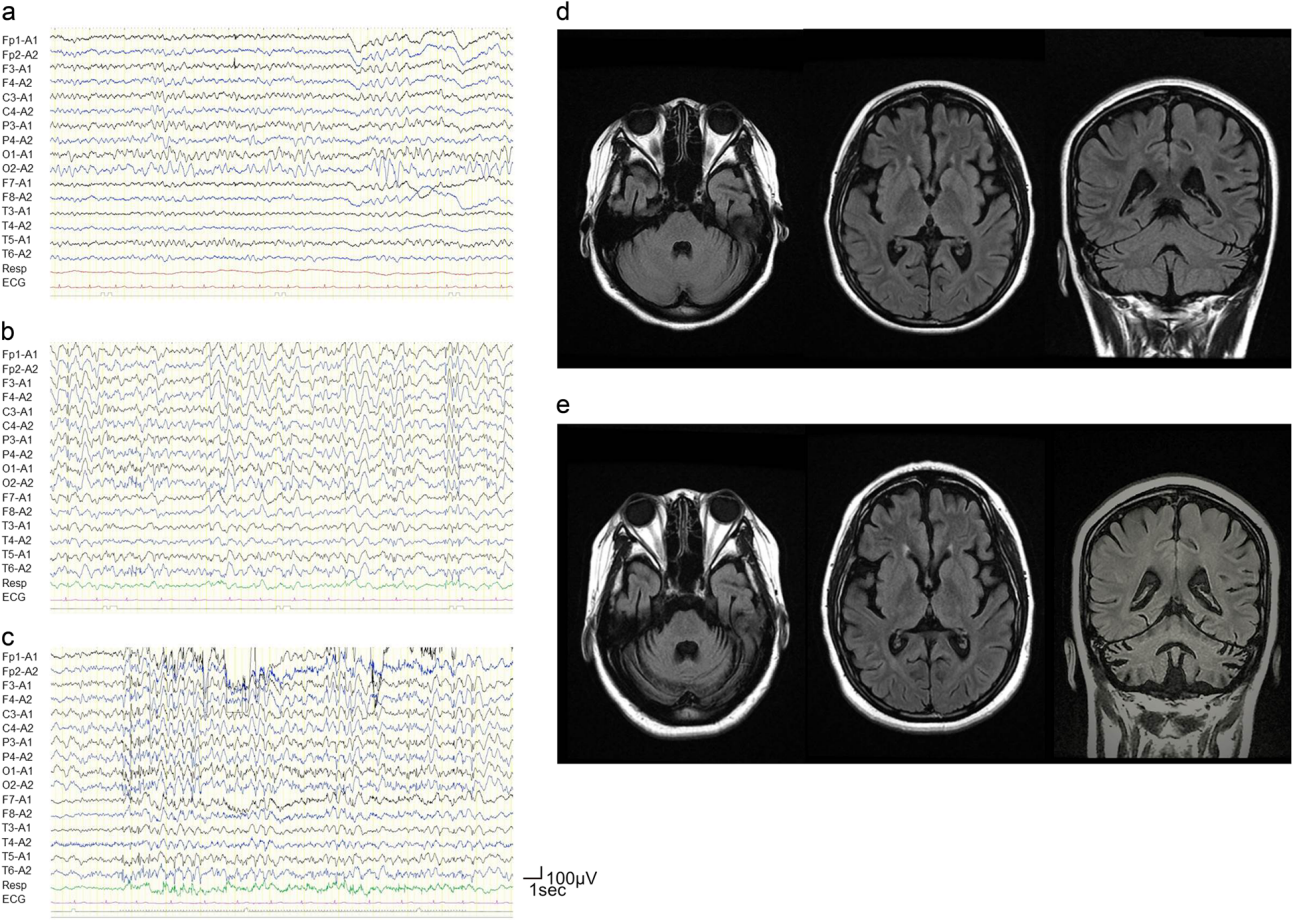
Electroencephalography and magnetic resonance imaging of the proband (III-4). **a** Continuous occipital slow waves were observed when she was 12 years old; **b** generalized spikes and slow waves, and **c** marked photosensitivity were observed when she was 14 years old. Brain magnetic resonance imaging (fluid attenuated inversion recovery) of the proband (III-4): **d** cerebral atrophy was prominent when she was 14 years old; **e** cerebellar atrophy appeared progressively when she was 17 years old

The proband was referred to our hospital at the age of 14 years. Myoclonic movement was occasionally observed in her fingers and eyelids. An examination of peripheral blood cells (PBCs) and sera showed insignificantly elevated hepatic enzyme levels. Urine and cerebrospinal fluid test results were normal. EEGs showed slow background activity, frequent generalized slow waves, and generalized and focal spikes (Fig. 2b). Bilateral diffuse slow waves intermingled with spikes were easily induced by intermittent photic stimulation (Fig. 2c). Fundoscopy revealed no abnormality. A somatosensory evoked potential (SEP) analysis showed a giant SEP. A brain magnetic resonance imaging scan showed moderate cerebral and cerebellar atrophy (Fig. 2d). These electro-clinical findings suggested LD, but Lafora bodies were not found by palm skin or rectal mucosal biopsy. We sent genomic DNA isolated from her PBCs to a company for analyses of the *EPM2A* and *NHLRC1* genes. Upon analysis, the company reported no variants in either *EPM2A* or *NHLRC1*. Furthermore, we excluded the following diseases: Gaucher disease by a glucocerebrosidase enzyme assay in fibroblasts and the absence of Gaucher cells in the bone marrow; Unverricht–Lundborg disease and Dentato-rubro-pallido-luysian atrophy by genetic examinations; and mitochondrial disease by the lactate and pyruvate levels in the serum and cerebrospinal fluid.

At the age of 16 years, the proband became bedridden. She continuously had focal and generalized myoclonus and seizures despite combination therapy with sodium valproate, clonazepam, lamotrigine, and topiramate. Brain MRI showed progression of cerebellar atrophy when she was 17 years old (Fig. 2e).

The younger sister of the proband (Fig. 1a; III-5) experienced a similar GTC following visual hallucinations and myoclonus since the age of 11 years. She was also of normal intelligence before disease onset, and memory disturbance and ataxia appeared a few years after onset. The subsequent onset in this younger sister strongly suggested that the two sisters suffered from an inherited PME.

When the proband was 17 years old and her younger sister was 15 years old, we performed whole-exome sequencing. The results showed that both patients had a homozygous frameshift variant in the *NHLRC1* gene (Fig. 1b), NM_198586.2 (NHLRC1):c.898del p. [Ser300Valfs*13], resulting in the substitution of the Serine300 residue with Valine followed by a premature stop codon at position 312. Sanger sequencing confirmed that their parents had a heterozygous NM_198586.2(NHLRC1):c.898del: p. [Ser300Valfs*13] variant. We requested re-examination of *NHLRC1* from the company that performed the original genetic analysis, and the variant was confirmed. The variant was not found in the Exome Variant Server database (http://evs.gs.washington.edu/EVS/), the Genome Aggregation Database (gnomAD) (http://gnomad.broadinstitute.org), or in our in-house 575 control exomes. The same variant had been reported by Singh et al.^4^; therefore, it was classified as pathogenic in accordance with the American College of Medical Genetics and Genomics variant classification guidelines^5^.

Considering the clinical symptoms and the results of genetic examinations, we diagnosed both patients with LD due to a homozygous frameshift variant in the *NHLRC1* gene.

The siblings exhibited the typical clinical characteristics of LD. Their PME with occipital seizures and photosensitivity appeared during their teenage years. Their intellectual and motor functions declined rapidly from the onset. Lafora bodies, a characteristic feature of LD showing deposition of abnormal glycogen in the cytoplasm, were not found in our cases, and definitive diagnosis was performed by detection of the *NHLRC1* variant. The prevalence of *EPM2A* or *NHLRC1* variants in classical LD patients was much higher (93%) than the detection rate of Lafora bodies (67%)^6^. Therefore, mutation screening of the *EPM2A* and *NHLRC1* genes is an effective and recommended method for the diagnosis of LD.

High allelic heterogeneity has been observed for LD^3^. It suggests that the variants occur from single events in most cases. On the other hand, several recurrent variants in the *NHLRC1* gene, such as the missense variant c.76T>A: p.Cys26Ser (rs28940575, Allele Frequency (AF) in gnomAD = 4.529 × 10^−6^) in French-Canadians, the frameshift variant c.468_469delAG: p.Gly158Argfs*17 (rs781300542, AF = 1.226 × 10^−5^) in Omani, the frameshift variant c.1049_1050del: p.Glu350Glyfs*41 (not registered in gnomAD) in the Serbian/Montenegrin and the missense variant c.205C>G:p.Pro69Ala (rs28940576, AF = 9.33 × 10^−5^) in Mediterranean countries, were reported^3,7,8^. These variants are very rare, but each variant was frequently found in LD families living in a specific region, suggesting a founder effect.

Previous studies described that *NHLRC1* is the common causative gene in Japanese LD patients, and five missense variants and a single base-pair deletion leading to a frameshift variant have been identified^4^. None of these variants are shared with the other five families in previous reports. The NM_198586.2(NHLRC1):c.898del:p.[Ser300Valfs*13] variant found in our patients has not yet been registered in public databases; however, the same homozygous variant was found in another Japanese LD patient born to non-consanguineous parents^4^. Our data suggest that the NM_198586.2 (NHLRC1):c.898del:p.[Ser300Valfs*13] variant may originate from the same ancestor in the limited northern Kyusyu districts, but further analyses are needed to confirm the founder effect of the c.898del variant in the limited districts.

In conclusion, we found a homozygous *NHLRC1* variant in Japanese LD siblings without consanguineous marriage. The genetic analysis was a minimally invasive and highly sensitive diagnostic method. Therefore, it should be recommended as the first step for LD diagnosis instead of the histological examination.

## Data Availability

The relevant data from this Data Report are hosted at the Human Genome Variation Database at 10.6084/m9.figshare.hgv.2327.
